# Improved Manta Ray Foraging Optimization for PID Control Parameter Tuning in Artillery Stabilization Systems

**DOI:** 10.3390/biomimetics10050266

**Published:** 2025-04-26

**Authors:** Xiuye Wang, Xiang Li, Qinqin Sun, Chenjun Xia, Ye-Hwa Chen

**Affiliations:** 1 School of Mechanical Engineering, Nanjing University of Science and Technology, Nanjing 210094, China; xiuyewang@njust.edu.cn (X.W.); lixiang123@njust.edu.cn (X.L.); xiachenjun@njust.edu.cn (C.X.); 2 School of Energy and Power Engineering, Nanjing University of Aeronautics and Astronautics, Nanjing 210016, China; 3 The George W. Woodruff School of Mechanical Engineering, Georgia Institute of Technology, Atlanta, GA 30332, USA; yehwa.chen@me.gatech.edu

**Keywords:** improved manta ray foraging optimization, artillery stabilization control systems, PID control parameter tuning, Lévy flight

## Abstract

In this paper, an Improved Manta Ray Foraging Optimization (IMRFO) algorithm is proposed to address the challenge of parameter tuning in traditional PID controllers for artillery stabilization systems. The proposed algorithm introduces chaotic mapping to optimize the initial population, enhancing the global search capability; additionally, a sigmoid function and Lévy flight-based dynamic adjustment strategy regulate the selection factor and step size, improving both convergence speed and optimization accuracy. Comparative experiments using five benchmark test functions demonstrate that the IMRFO algorithm outperforms five commonly used heuristic algorithms in four cases. The proposed algorithm is validated through co-simulation and physical platform experiments. Experimental results show that the proposed approach significantly improves control accuracy and response speed, offering an effective solution for optimizing complex nonlinear control systems. By introducing heuristic optimization algorithms for self-tuning artillery stabilization system parameters, this work provides a new approach to enhancing the intelligence and adaptability of modern artillery control.

## 1. Introduction

The control problem of artillery stabilization systems has long been a research focus. Early stabilization systems primarily relied on mechanical stabilizers. Traditional control strategies such as PID controllers have been widely applied in artillery stabilization systems. However, with the continuous advancement of artillery technology, control strategies for artillery stabilization have evolved to meet increasingly complex system demands. Due to the complexity of modern artillery systems, particularly those driven by electro-hydraulic actuators, control performance is influenced not only by mechanical design but also by nonlinear factors and external disturbances [1]. These factors significantly impact the stability accuracy of artillery in real combat environments. Moreover, the presence of uncertainties such as mechanical collisions, structural deformations, and friction forces that are difficult to model accurately together with mechanical wear over time make PID parameter tuning more challenging [2]. Therefore, determining appropriate control parameters that can adapt to complex and dynamically changing environments remains an urgent problem to be addressed in artillery stabilization systems.

Various control approaches have been proposed to address the challenges posed by strong nonlinearities and time-varying parameters in artillery stabilization systems, including neural network control, adaptive control, and fuzzy inference control [3,4,5,6,7]. Wang et al. [8] introduced a variable-structure wavelet neural network optimized using an adaptive differential evolution algorithm, improving both system accuracy and response speed. Ma et al. [9] proposed an adaptive robust feedback control strategy and verified its effectiveness through co-simulation. Rafiei et al. [10] incorporated fuzzy logic into a radial basis function (RBF) neural network for online optimization of control parameters. These studies primarily focus on model-based online optimization of control parameters to ensure basic accuracy under complex operating conditions.

Online tuning strategies have proven effective in artillery control systems, but often rely on real-time computation and high-fidelity models, which can increase implementation costs and hardware complexity. This challenge has led to the exploration of offline parameter tuning approaches [11,12], which utilize precalibrated optimization to minimize computational burdens during operation. Offline optimization has been widely explored in areas such as robotics and power systems, where it has shown the potential to enhance system performance and efficiency. To improve control accuracy while tuning parameters in a model-free environment, some researchers have turned to intelligent optimization algorithms for offline PID parameter optimization. For instance, Kong et al. [13] proposed an improved Dung Beetle Optimizer (DBO) for self-coupling PID parameter tuning. Rodrigues et al. [14] developed an enhanced Particle Swarm Optimization (PSO) algorithm to optimize PID parameters in automatic voltage regulators, while Azeez et al. [15] introduced an Artificial Bee Colony (ABC) algorithm with an adaptive learning strategy for optimizing control parameters in robotic arms.

The Manta Ray Foraging Optimization (MRFO) algorithm [16], first proposed in 2020, is an innovative bio-inspired swarm intelligence algorithm. This algorithm draws inspiration from the unique foraging behavior of manta rays, which utilize chain foraging, spiral foraging, and somersault foraging strategies to enhance their search capabilities. The manta ray’s flattened pectoral fins and broad dorsal fins offer exceptional maneuverability, enabling rapid directional changes and adjustments in posture. These foraging behaviors are effectively emulated by the MRFO algorithm, which is distinguished by its simplicity, minimal parameter requirements, and robust abilities in both global and local search. It has been widely applied to solve various optimization problems, including power systems, mechanical design, image segmentation, and path planning [17,18,19,20]. For instance, Ma et al. [21] proposed a two-strategy enhanced MRFO for image segmentation, demonstrating the algorithm’s strong adaptability and competitiveness in visual data analysis. Similarly, Adamu et al. [22] employed MRFO for hyperparameter optimization in skin cancer classification, revealing its potential in complex biomedical signal processing tasks. These applications reflect the potential and applicability of the MRFO algorithm in addressing diverse optimization challenges across multiple fields. Despite its advantages, the standard MRFO algorithm faces challenges, particularly in high-dimensional function optimization problems, where it tends to converge to local optima and exhibits slow convergence rates. These limitations hinder the algorithm’s performance in complex optimization tasks such as parameter tuning in systems with intricate dynamics.

To further enhance the performance of the artillery stabilization control system in a model-free setting, this paper proposes an Improved Manta Ray Foraging Optimization (IMRFO) algorithm that integrates multiple optimization strategies for parameter tuning. First, circle mapping, Lévy flight, and the sigmoid function are introduced to enhance the global search capability of the traditional MRFO algorithm. Then, the effectiveness of the proposed optimization method is validated using five benchmark test functions. Finally, the algorithm’s effectiveness and feasibility are demonstrated through co-simulation and physical system verification.

This paper makes three significant contributions. First, an Improved Manta Ray Foraging Optimization (IMRFO) algorithm incorporating circle chaotic mapping, Lévy flight, and a sigmoid function-based dynamic adjustment strategy is proposed to enhance the global search capability and convergence speed of the traditional MRFO algorithm. Second, a PID control parameter optimization method based on the IMRFO algorithm is designed for artillery stabilization systems. This method effectively addresses the challenges posed by strong nonlinearities and time-varying parameters, significantly improving the system’s stability and response performance. Third, the effectiveness and feasibility of the proposed method are validated through simulations and physical system experiments, demonstrating its potential as a reliable and efficient solution for complex control systems.

## 2. MRFO Algorithm

The MRFO strategy consists of a swarm intelligence optimization algorithm inspired by the foraging behavior of manta rays. Manta rays are highly adaptive and exhibit unique behaviors that allow them to efficiently locate food sources. In nature, their foraging process demonstrates adaptability, cooperative behavior, and strong local search capabilities, which inspired the development of the MRFO algorithm. The foraging behavior of manta rays can be classified into three types: chain foraging, spiral foraging, and somersault foraging.

### 2.1. Chain Foraging

**Figure 1:**
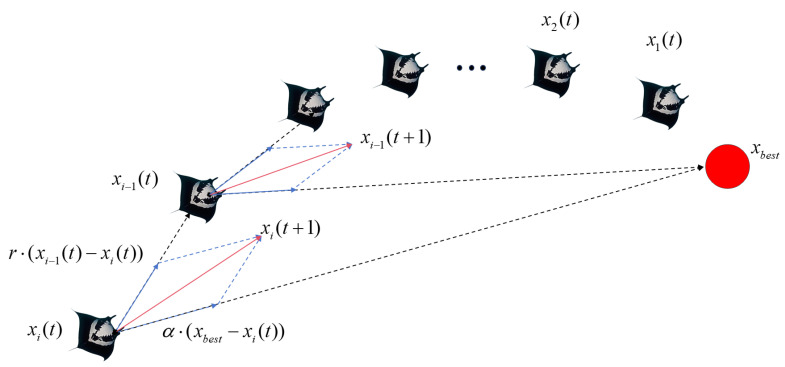
Chain foraging strategy diagram.

The chain foraging strategy simulates the cooperative behavior of manta ray groups when searching for food. Each manta ray adjusts its direction not only based on the local food concentration but also by referencing the position of the manta ray ahead of it. This creates a “chain” in which each individual follows the one in front, continuously moving toward the food source and the leading individuals. This cooperative approach enhances the efficiency of the group in converging toward the optimal location while improving the collective search capability. The mathematical model of chain foraging is as follows:

(1) $$x_{i}^{d}\left(t+1\right)=\left\{\begin{array}{cc} {x_{i}}^{d}\left(t\right)+r\left({x_{best}}^{d}\left(t\right)-{x_{i}}^{d}\left(t\right)\right)+\alpha \left({x_{best}}^{d}\left(t\right)-{x_{i}}^{d}\left(t\right)\right), & i=1,\\ {x_{i}}^{d}\left(t\right)+r\left({x_{i-1}}^{d}\left(t\right)-{x_{i}}^{d}\left(t\right)\right)+\alpha \left({x_{best}}^{d}\left(t\right)-{x_{i}}^{d}\left(t\right)\right), & i\geq 2, \end{array}\right.$$

(2) $$\alpha =2r\sqrt{\left|\log(r)\right|},$$

where ${x_{i}}^{d}\left(t\right)$ is the position of the *i*-th individual in the *d*-th dimension at the *t*-th iteration, *r* is a random number in [0, 1], ${x_{best}}^{d}\left(t\right)$ is the individual with the highest fitness value in the current iteration, which can be understood as the current food position, and α is the chain foraging weight factor, which determines whether the current individual tends to optimize towards the previous individual or the historically best individual in this iteration. Figure 1 illustrates the behavioral strategy of chain foraging.

### 2.2. Spiral Foraging

**Figure 2:**
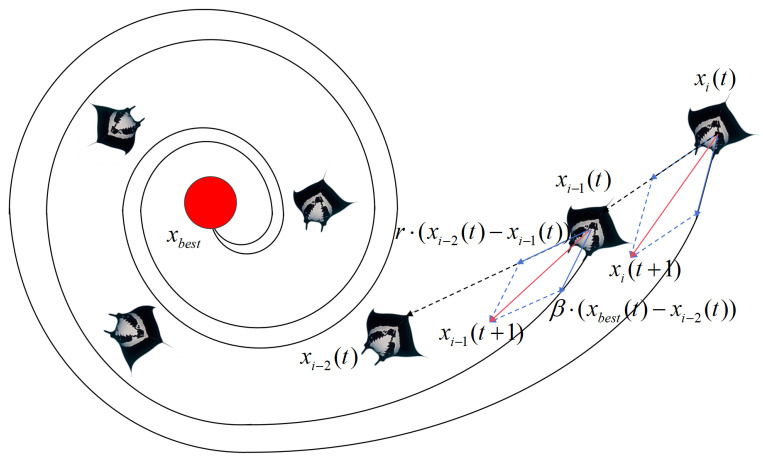
Spiral foraging strategy diagram.

Compared to chain foraging, spiral foraging focuses more on global exploration. In chain foraging, the movement of manta rays is primarily determined by the positions of the preceding individuals and the current food source, leading to strong local search characteristics due to the close connections among individuals. This means that most of the searching and adjustments occur within a limited area. In contrast, spiral foraging enables manta rays to move along spiral trajectories toward the food source, allowing them to explore a broader space. Individuals in spiral foraging are influenced not only by the preceding individuals but also by the spiral motion, thereby expanding the overall search area. This approach helps to prevent premature convergence and improves the algorithm’s ability to escape local optima. The mathematical model of spiral foraging is as follows:

(3) $$x_{i}^{d}\left(t+1\right)=\left\{\begin{array}{cc} {x_{best}}^{d}\left(t\right)+r\left({x_{best}}^{d}\left(t\right)-{x_{i}}^{d}\left(t\right)\right)+\beta \left({x_{best}}^{d}\left(t\right)-{x_{i}}^{d}\left(t\right)\right), & i=1,\\ {x_{best}}^{d}\left(t\right)+r\left({x_{i-1}}^{d}\left(t\right)-{x_{i}}^{d}\left(t\right)\right)+\beta \left({x_{best}}^{d}\left(t\right)-{x_{i}}^{d}\left(t\right)\right), & i\geq 2, \end{array}\right.$$

(4) $$\beta =2e^{\frac{r_{1}\left(T-t+1\right)}{T}}\sin\left(2\pi r_{1}\right),$$

where β is the weight factor for spiral search and *T* is the maximum number of iterations. Figure 2 depicts spiral foraging strategy.

In the spiral search strategy, each individual’s search behavior revolves around the current optimal solution, ensuring refined exploration in local regions. To enhance global search capability, a new reference position is randomly selected to drive individuals away from the existing optimal solution, encouraging exploration of new areas.

(5) $$x_{i}^{d}\left(t+1\right)=\left\{\begin{array}{cc} {x_{rand}}^{d}\left(t\right)+r\left({x_{rand}}^{d}\left(t\right)-{x_{i}}^{d}\left(t\right)\right)+\beta \left({x_{rand}}^{d}\left(t\right)-{x_{i}}^{d}\left(t\right)\right), & i=1,\\ {x_{rand}}^{d}\left(t\right)+r\left({x_{i-1}}^{d}\left(t\right)-{x_{i}}^{d}\left(t\right)\right)+\beta \left({x_{rand}}^{d}\left(t\right)-{x_{i}}^{d}\left(t\right)\right), & i\geq 2, \end{array}\right.$$

(6) $${x_{rand}}^{d}=Lb^{d}+r\left(Ub^{d}-Lb^{d}\right),$$

where ${x_{rand}}^{d}$ represents a randomly selected position within the current search space, while $Lb^{d}$ and $Ub^{d}$ respectively denote the lower and upper bounds of the current position space.

### 2.3. Somersault Foraging

**Figure 3:**
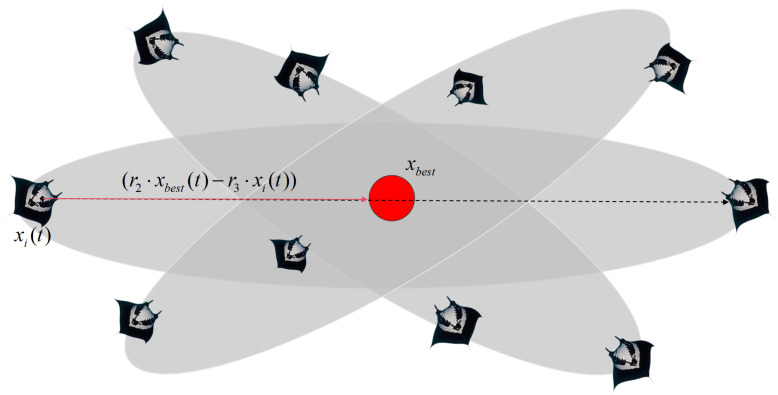
Somersault foraging strategy diagram.

In the somersault foraging behavior, manta rays adjust their positions by rolling around the current optimal solution. This movement pattern allows them to explore new locations in a manner similar to a somersault. By continuously updating their positions relative to the best solution found so far, the individuals enhance their ability to refine the search and improve the optimization process. The mathematical model of somersault foraging is as follows:

(7) $${x_{i}}^{d}\left(t+1\right)={x_{i}}^{d}\left(t\right)+S\left(r_{2}{x_{best}}^{d}-r_{3}{x_{i}}^{d}\left(t\right)\right)$$

where *S* is the roll factor that determines the rolling range and where $r_{2}$ and $r_{3}$ are random numbers in [0, 1]. Figure 3 illustrates the somersault foraging strategy.

## 3. Improved MRFO Algorithm

### 3.1. Circle Chaotic Mapping for Population Initialization

In artillery stabilization systems, the complexity of simulations and high time cost of physical experiments often limit the feasible population size in optimization algorithms. Under such constraints, the standard MRFO algorithm’s reliance on random initialization can lead to insufficient population diversity, hindering comprehensive exploration of the solution space. To address this, we introduce circle chaotic mapping for population initialization. This method generates initial solutions with better uniformity and ergodicity compared to random initialization, thereby enhancing the algorithm’s ability to explore the search space comprehensively and avoid premature convergence. By improving the diversity of the initial population, the chaotic mapping strategy effectively mitigates the limitations of standard MRFO during the complex high-dimensional optimization tasks inherent in artillery stabilization systems.

Chaos mapping is a method for generating random behavior based on deterministic systems. It is capable of producing sequences with ergodicity and non-repetition within a finite range [23]. In this paper, circle chaos mapping is used for population initialization. Circle chaos mapping is a chaotic mapping method based on trigonometric functions and modular arithmetic, and its mathematical expression is as follows:

(8) $$x_{k+1}=\;\mathrm{mod}\;\left(x_{i}+b-\frac{a}{2\pi }\sin\left(2\pi x_{i}\right),1\right),$$

(9) $${x_{i}}^{d}=Lb^{d}+{x_{k+1}}^{d}\left(Ub^{d}-Lb^{d}\right),$$

where the value range of the circle chaos mapping sequence is [0, 1] and where *a* and *b* are control parameters. To ensure the randomness and uniformity of the chaos mapping, *a* and *b* are generally set to values around 0.5 and 0.2, respectively. The steps for population initialization using circle chaotic mapping are as follows:

**Step 1:**  Set the population size *N* and dimension *D*, initializing the first individual ${x_{1}}^{j}\in \left(0,1\right)$ in each dimension. The initial values of *i* and *j* are 1.
**Step 2:**  Initialize the control parameters as follows: a∈(0.45,0.55), b∈(0.2,0.25).
**Step 3:**  With i=i+1, generate ${x_{i}}^{j}$ according to Equation (8).
**Step 4:**  If *i* is greater than *N*, proceed to Step 5; otherwise, return to Step 3.
**Step 5:**  With j=j+1, check whether *j* is greater than *D*. If no, then return to Step 2 and set i=1; if yes, then the initialization is complete and the initial population positions are output according to Equation (9).

### 3.2. Sigmoid Function-Based Strategy Selection Factor

In the MRFO algorithm, each iteration alternates between chain and spiral foraging based on a strategy selection factor λ(t), which is typically fixed in standard implementations. However, this approach may not fully address the complexities of artillery stabilization systems, where the dynamics are highly nonlinear and influenced by numerous factors such as barrel vibrations, projectile characteristics, and environmental conditions. In such systems, an adaptive strategy is crucial; early iterations should emphasize global exploration in order to thoroughly search the solution space, while later iterations should focus on local exploitation in order to fine-tune the solution near the optimal region. To balance global search and local exploitation, this paper proposes a strategy selection factor based on the sigmoid function, allowing the algorithm to adaptively adjust in different phases.

The sigmoid function is a commonly used nonlinear function, and its mathematical expression is as follows:

(10) $$S\left(x\right)=\frac{1}{1+e^{-k(x-x_{0})}}$$

where *x* is the input variable, *k* is the parameter that controls the slope of the function, and $x_{0}$ is the center point of the function. In the improved MRFO algorithm, the sigmoid function is used to adjust the strategy selection factor. Its mathematical expression is

(11) $$\lambda \left(t\right)=\lambda_{\min}+\frac{\lambda_{\max}-\lambda_{\min}}{1+e^{-k(t-0.5T)/T}},$$

where λ(t) is the strategy selection factor for the t-th iteration, $\lambda_{\min}$ and $\lambda_{\max}$ are the minimum and maximum values of the strategy selection factor, respectively, and *T* is the maximum number of iterations. By setting $\lambda_{\min}=0.25$, $\lambda_{\max}=0.75$, the algorithm tends to favor spiral foraging during the early iterations, which helps it to quickly explore the solution space and avoid becoming trapped in local optima. In the later stages of the iteration, the focus shifts to chain foraging, which enables fine-tuning around the optimal solution and improves convergence accuracy.

### 3.3. Lévy Flight-Integrated Somersault Foraging

The MRFO algorithm also employs somersault foraging, in which individuals oscillate around the current optimal solution to enhance local exploration. However, using a fixed oscillation range can result in premature convergence to local optima. To address this limitation, the proposed algorithm incorporates Lévy flight step sizes during the somersault phase, introducing random perturbations that expand the search range and improve the algorithm’s global exploration capability. This enhancement is particularly beneficial for optimizing complex systems such as artillery stabilization, where the search space is vast and traditional optimization methods may struggle to escape local minima. By integrating Lévy flight, the ability of the MRFO algorithm to explore diverse solutions is enhanced, leading to more robust and effective optimization outcomes in challenging engineering applications.

Lévy flight is a stochastic walk strategy based on a heavy-tailed distribution, where most step sizes are short but long steps occasionally occur with a small probability [24]. This characteristic allows Lévy flight to balance local exploitation and global exploration. Its mathematical expression is

(12) $$L\left(s\right)=\frac{\mu }{{\left|\nu \right|}^{1/\beta }},$$

(13) $$\left\{\begin{array}{c} \sigma_{\mu }={\left[\frac{\Gamma (1+\beta )\sin(\pi \beta /2)}{\Gamma \left(\left(1+\beta \right)/2\right)2^{(\beta -1)/2}\beta }\right]}^{1/\beta },\\ \sigma_{\nu }=1, \end{array}\right.$$

where Γ is the Gamma function, L(s) is the Lévy flight step length, and β is the Lévy exponent, which determines the probability of large step lengths. Usually, β=1.5 is set and both μ and ν follow a normal distribution such that

(14) $$\mu ∼N(0,\sigma_{\mu }^{2}),$$

(15) $$\nu ∼N(0,\sigma_{\nu }^{2}).$$

**Figure 4:**
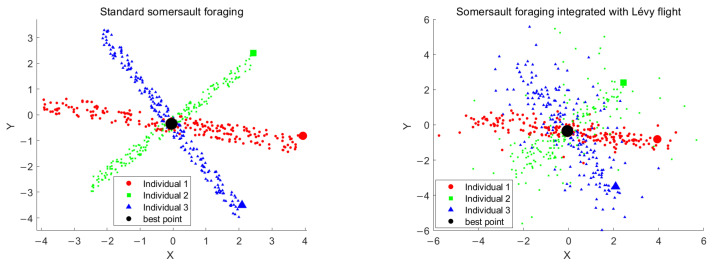
Distribution of individuals in two-dimensional space after somersault foraging.

To enhance the search efficiency of the somersault foraging strategy, the Lévy flight step length is introduced into the position update process and Equation (7) is updated as follows:

(16) $${x_{i}}^{d}\left(t+1\right)={x_{i}}^{d}\left(t\right)+S\left(r_{2}{x_{best}}^{d}-r_{3}{x_{i}}^{d}\left(t\right)\right)+kL\left(s\right)\left({x_{best}}^{d}-{x_{i}}^{d}\left(t\right)\right)$$

where *k* is the Lévy flight step length control factor, which helps to avoid excessive influence of short step lengths on local fine-tuning. Figure 4 shows the position distribution of three individuals after 200 somersaults.

Figure 4 shows the somersault foraging strategy in the standard MRFO algorithm, where the individuals are concentrated within the rolling region after somersaulting. In contrast, using the improved somersault foraging strategy with the random step length characteristic of Lévy flight expands the search space. Most individuals remain concentrated in the rolling region near the current optimal solution for fine-grained search, while a small portion of individuals jump out of the current region through long steps to explore other solution spaces. This approach retains the efficiency of local exploitation while enhancing global exploration capability.

Our proposed improved MRFO algorithm is subsequently referred to as the IMRFO algorithm. The pseudocode for the IMRFO algorithm is shown in Algorithm 1.
**Algorithm 1** IMRFO Algorithm1:Initialization: Population size *N*, number of iterations *T*, upper bound Ub, lower bound Lb2:Generate initial population positions based on Equation (8), calculate fitness values of all individuals, and find the best solution $x_{best}$3:**repeat**4:   t←t+15:   **if** rand<λ(t) **then**6:     Spiral foraging7:     **if** t/T<rand **then**8:        Update positions of all individuals based on Equation (5)9:     **else**10:        Update positions of all individuals based on Equation (3)11:     **end if**12:   **else**13:     Chain foraging14:     Update positions of all individuals based on Equation (1)15:   **end if**16:   Update the best fitness value17:   **for** i=1*N* **do**18:     somersault foraging19:     Update positions of all individuals based on Equation (16)20:     Update the best fitness value again21:   **end for**22:**until** t≥T is no longer true**Output:** Best fitness value and position of the optimal individual

### 3.4. Algorithm Validation

To evaluate the performance of the proposed algorithm, five benchmark functions are used for assessment; $f_{1}$ and $f_{2}$ are unimodal test functions, while $f_{3}$, $f_{4}$, and $f_{5}$ are multimodal test functions. The unimodal test functions are primarily used to assess the convergence speed of the algorithm, whereas the multimodal test functions are chosen based on those areas where the standard IMRFO algorithm exhibits relatively weaker performance. This selection aims to further validate the proposed algorithm’s global optimization capability in complex problems. The benchmark functions are shown in Table 1.

We evaluated the IMRFO algorithm against five commonly used optimization algorithms, including the Manta Ray Foraging Optimization (MRFO) algorithm [25], Particle Swarm Optimization (PSO) algorithm [26], Grey Wolf Optimizer (GWO) algorithm [27], Whale Optimization Algorithm (WOA) [28], Ant Lion Optimizer (ALO) algorithm [29], and Covariance Matrix Adaptation Evolution Strategy (CMA-ES) [30]. For all algorithms, the population size was set to 100 with 2000 iterations, and each algorithm was run ten times.

The parameter settings for the comparison algorithms are shown in Table 2. In Particle Swarm Optimization (PSO), the inertia weight was decreased linearly from 0.9 to 0.4, gradually shifting the search focus from global to local exploration. The cognitive and social coefficients were both set to 1.5, ensuring a balanced influence between individual experience and social sharing. For the Grey Wolf Optimizer (GWO) and Whale Optimization Algorithm (WOA), the convergence control parameter *a* decreased linearly from 2 to 0, allowing for a smooth transition between the exploration and exploitation phases. In the Ant Lion Optimizer (ALO) algorithm, the random walk boundaries were adaptively adjusted based on iteration count and ant lion selection was performed via roulette wheel selection to maintain diversity. In the Covariance Matrix Adaptation Evolution Strategy (CMA-ES), the initial step size σ was set to one-third of the search range to provide a balanced initial exploration. The parent number μ was set to half of the population size to ensure sufficient diversity. The step control parameter $c_{s}$ and covariance matrix update rate $c_{1}$ were computed based on the problem dimension and effective parent number to maintain stability and efficient adaptation of the covariance matrix.

**Figure 5:**
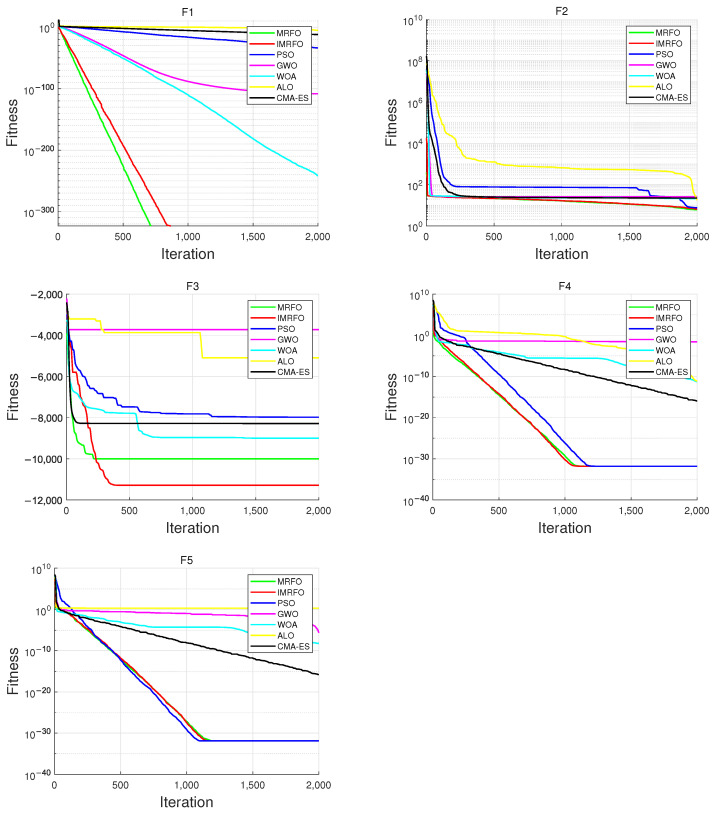
Convergence characteristics and comparison results of the six algorithms on the test functions.

The optimal and average values of each algorithm on the benchmark functions are presented in Table 3, while the convergence curves are shown in Figure 5. It can be observed that the convergence speed of IMRFO is slightly slower than that of MRFO for the unimodal functions $f_{1}$ and $f_{2}$, which is due to the updates in the somersault model. However, both algorithms quickly converge to the optimal solution, and the optimal solution and the mean optimal solution are significantly better than those of other algorithms. For multimodal function $f_{3}$, IMRFO outperforms MRFO in both convergence speed and accuracy, indicating that the improves the strategy selection factor enhances the global optimization capability in the early iterations and focuses more on local exploration in the later stages. For multimodal function $f_{4}$, IMRFO achieves the best optimal solution and mean value, with a mean that is significantly better than the other algorithms. This suggests that the improved somersault foraging mechanism can better escape local optima and enhance the stability of the algorithm. For multimodal function $f_{5}$, the MRFO algorithm was only able to escape local optima in two out of the ten optimization runs, while the proposed IMRFO algorithm was able to find the optimal solution multiple times, further proving the global optimization capability of the improved somersault foraging strategy.

The Wilcoxon Signed-Rank Test (WSRT) is used to assess statistical differences between two optimizers. The results for the ten runs across the various benchmark functions are presented in Table 4 and Table 5. The *p*-value indicates statistical significance, with values below 0.05 showing a significant difference; “W” represents the Wilcoxon test statistic, calculated as the smaller of the sums of ranks for positive and negative differences between paired observations; the “Result” column shows “+” for better performance by the first algorithm, “−” for the second, and “=” for no significant difference. The results indicate that IMRFO outperforms MRFO with significant differences (*p*-value < 0.05) in most cases, particularly for multimodal functions $f_{3}$ and $f_{5}$. IMRFO also shows better performance than the other algorithms on most functions. However, for function $f_{5}$, IMRFO shows no significant advantage over CMA-ES or PSO. Overall, the test results demonstrate that IMRFO generally outperforms the other algorithms, confirming its effectiveness.

## 4. Parameter Optimization of Artillery Stabilization Control System Based on IMRFO

### 4.1. Development of Co-Simulation Platform

To verify the effectiveness of the IMRFO algorithm in the PID controller of an artillery stabilization control system, a system model was established using a complex system modeling and simulation platform. Based on the system structural parameters shown in Table 6, the dynamic model of the artillery stabilization control system was built in a multibody dynamics simulation platform. The control system was designed in a control simulation environment using a PID controller, and the IMRFO algorithm was implemented in an intelligent optimization module to optimize the PID controller parameters.

**Figure 6:**
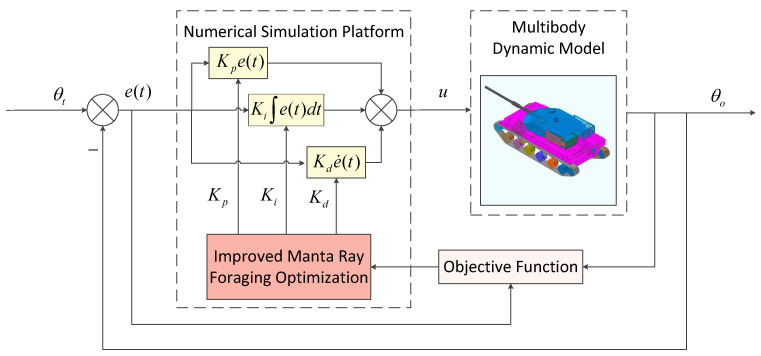
Block diagram of artillery stabilization control system parameter optimization based on IMRFO.

To achieve seamless coordination between the control system and the mechanical system, a co-simulation platform was established by integrating a multibody dynamics simulation platform with a numerical control system as illustrated in Figure 6. This setup enables real-time data exchange and computation between the control model and the artillery dynamics model. The control system processes the barrel elevation displacement state parameters obtained from the multibody dynamics model and computes the corresponding output torque, which is then fed back to drive the mechanical system. This approach ensures accurate and efficient interaction between the control strategy and the dynamic behavior of the artillery system.

### 4.2. Simulation Parameter Settings

Let the control parameters *P*, *I*, and *D* be $K_{p}$, $K_{i}$, and $K_{d}$, respectively. Through multiple simulation verification runs, it was found that the control system tends to diverge when the controller parameters are too large or too small. To prevent ineffective searches by the algorithm, the search ranges for the control parameters were set to $K_{p}\in \left[1,5000\right]$, $K_{i}\in \left[0.5,2000\right]$, and $K_{d}\in \left[0.5,300\right]$.

In the IMRFO algorithm, the population size was set to *N* and the maximum number of iterations was *T*. Larger values of *N* and *T* improve convergence accuracy, but significantly increase training time. Considering these factors, *N* and *T* were chosen as N=10 and T=20.

Considering the control requirements of high tracking accuracy and fast response speed, the objective function is defined as

(17) $$J=\omega_{1}\int_{0}^{t_{ss}}\left|e(t)\right|dt+\omega_{2}\int_{0}^{t_{ss}}t\left|e(t)\right|dt,$$

where $t_{ss}$ is the simulation time, $\omega_{1}$ and $\omega_{2}$ are the weight coefficients for response speed and tracking accuracy, respectively, and e(t) represents the difference between the actual system output and the desired output, with $t_{ss}=10$, $\omega_{1}=5$, and $\omega_{2}=1$.

To verify the effectiveness and accuracy of the proposed IMRFO algorithm in the artillery stabilization control system, a comparative analysis was performed between the IMRFO and WOA algorithms, both of which performed well in benchmark function tests. Sinusoidal were used to validate the dynamic control performance of the artillery elevation stabilization system, while step inputs were used to validate its static performance. The desired elevation angle motion trajectories under both conditions were as follows:

(18) θ(t)=0.15+0.1sin(12πt)rad,

(19) θ(t)=0.4rad.

In addition, to better evaluate the control performance of the controller, we used the Integral Absolute Error (IAE) and Integral Time-weighted Absolute Error (ITAE) as our performance metrics. Among these, the IAE reflects the cumulative error of the system during the entire operation, while the ITAE focuses more on the error performance over a longer period of time. The specific expressions are as follows:

(20) $$\mathrm{IAE}=\int_{0}^{t_{ss}}\left|e\left(t\right)\right|dt,$$

(21) $$\mathrm{ITAE}=\int_{0}^{t_{ss}}t|e(t)|dt.$$

### 4.3. Simulation Results

**Figure 7:**
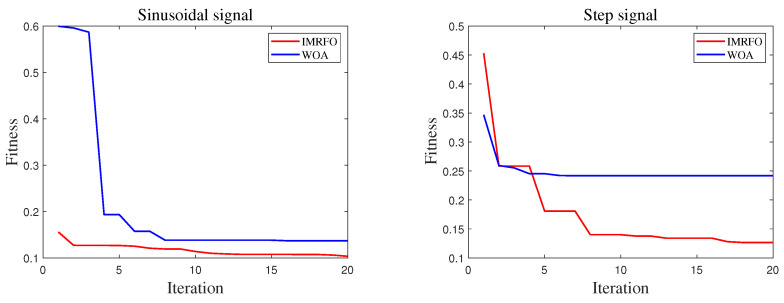
Variation curve of the optimal individual fitness value.

**Figure 8:**
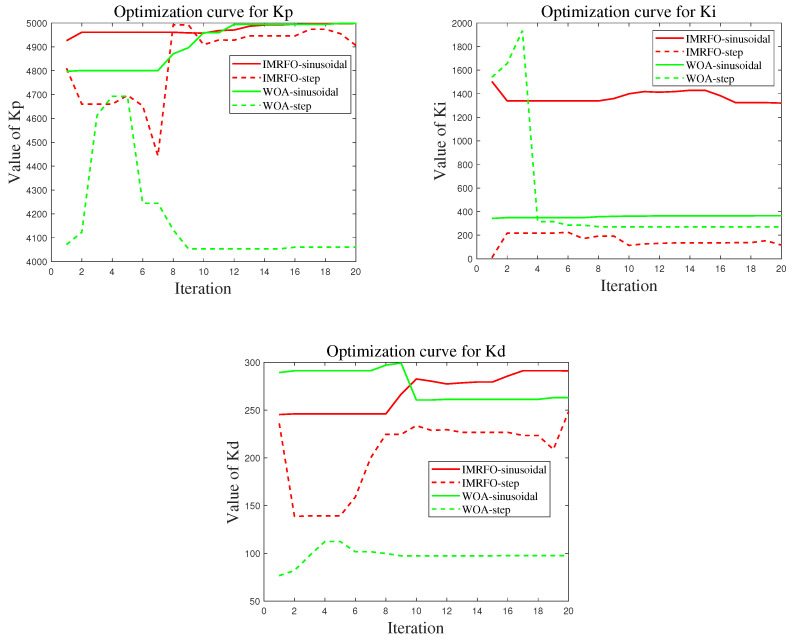
Control parameter optimization curve.

**Figure 9:**
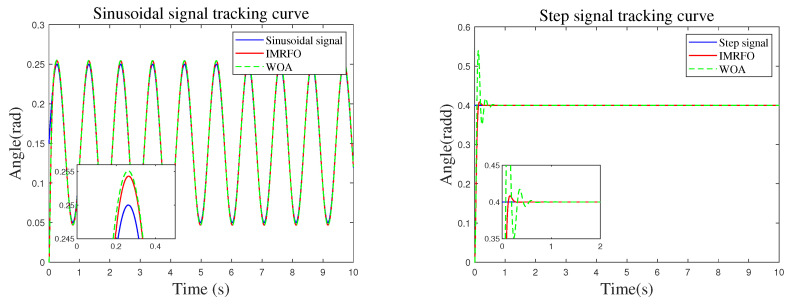
Tracking curve under two types of signals.

**Figure 10:**
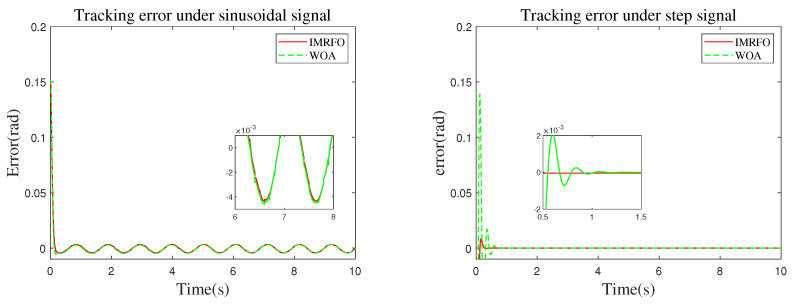
Tracking error under two types of signals.

The simulation results are shown in Figure 7, Figure 8, Figure 9 and Figure 10. In these figures, the two graphs in Figure 7 represent the fitness value convergence curves of the IMRFO algorithm under sinusoidal and step signals. From the simulation results, it can be seen that the IMRFO algorithm demonstrates superior performance over the WOA algorithm in both scenarios. Additionally, the IMRFO algorithm achieves relatively good results in the early stages of training due thanks to its incorporation of chaotic mapping-based population initialization.

The three graphs in Figure 8 present the iteration curves of the control parameters. After multiple iterations, the global optimal solution of the IMRFO algorithm under sinusoidal signal input is $K_{p}=4997.8$, $K_{i}=799.8$, $K_{d}=248.9$, while the global optimal solution of the WOA is $K_{p}=4999.9$, $K_{i}=365.1$, $K_{d}=263.4$. Under step signal input, the global optimal solution of the IMRFO algorithm is $K_{p}=4906.4$, $K_{i}=116.3$, $K_{d}=248.5$, while the global optimal solution of the WOA is $K_{p}=4060.6$, $K_{i}=270.5$, $K_{d}=97.6$.

**Figure 11:**
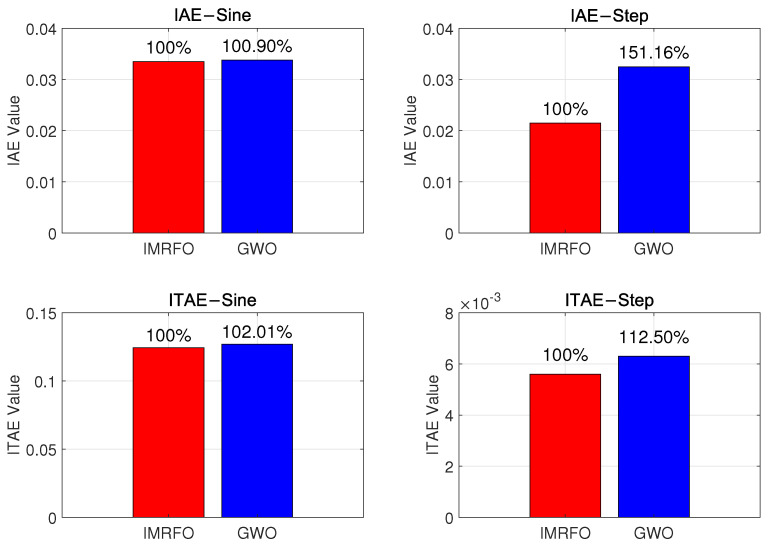
Comparison of performance metrics between the optimal parameters obtained by the IMRFO and GWO algorithms.

Figure 9 and Figure 10 compare the control system’s input–output and error, respectively. The controller performance is shown in Figure 11. Under sinusoidal input, the IMRFO and GWO algorithms perform similarly, with IMRFO slightly outperforming GWO in terms of IAE and ITAE values. However, under step output, IMRFO significantly outperforms GWO, with GWO’s IAE value being 51% higher than that of IMRFO and its ITAE value being 11% higher. Additionally, IMRFO converges within 0.3 s without significant oscillation; although the GWO algorithm has a lower steady-state error than the IMRFO algorithm, it has a slower response time and excessive overshoot.

### 4.4. Physical Validation

To validate the control performance of the PID controller parameters optimized by the IMRFO algorithm in an actual artillery stabilization system, a physical verification test was conducted. The primary objective of the test was to evaluate whether the optimized control parameters could outperform the empirical parameters in real-world applications and to further verify the practicality and reliability of the algorithm.

The physical verification was conducted on a semi-physical co-simulation platform integrating mechanical structure and electrical control. The test platform was provided by the China North Vehicle Research Institute. It was configured to focus on the vertical stabilization subsystem, with the performance of a key electromechanical actuator evaluated under different parameter settings. The mechanical subsystem includes key components such as a cradle, barrel, electric actuator, and adjustable hinges. The electrical control subsystem supports closed-loop control, comprising a host computer and a real-time processor from the mainstream DSP series. The system operates within a local network environment, allowing for real-time transmission of control commands and feedback signals.

Sensors such as a tilt sensor, rotary transformer, and eddy current probe were used to monitor the absolute and relative angular positions as well as the structural clearances. The control algorithm was developed in a mainstream embedded development environment and deployed to the DSP. Simulink-based models were compiled and downloaded to the real-time hardware, with experiment management handled via dSPACE ControlDesk. During the experiment, the system was powered up in a fixed sequence and real-time execution was initiated by switching to animation mode. The entire setup ensured that control strategies could be validated under realistic electromechanical coupling conditions, supporting effective evaluation of algorithm performance.

The experimental platform was configured to investigate the optimization of PI parameters within the velocity loop of the vertical stabilization system. The empirically determined parameters of $K_{p}=0.1$ and $K_{i}=10$ were used as the benchmark for comparison. To prevent system oscillation and divergence caused by improper control parameters, the optimization search range was set to $K_{p}\in \left[0.02,0.11\right]$, $K_{i}\in \left[1,14\right]$.

During the experiment, the reference signals included a sinusoidal signal (frequency 0.3 Hz, amplitude approximately 1°/s) and a step signal (output value of approximately 1°/s). The IMRFO algorithm was used to optimize the PI parameters with the objective of minimizing the tracking error of the control system for the sinusoidal signal, thereby enhancing the system’s dynamic response and stability.

**Figure 12:**
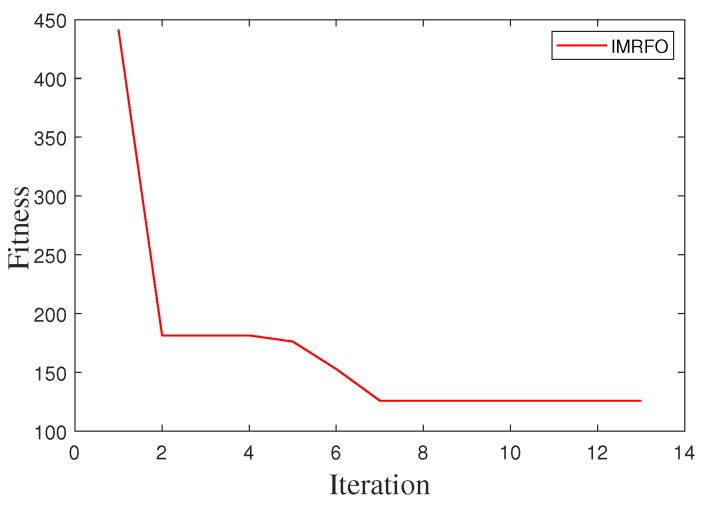
Fitness variation curve of the IMRFO algorithm on the physical turret.

**Figure 13:**
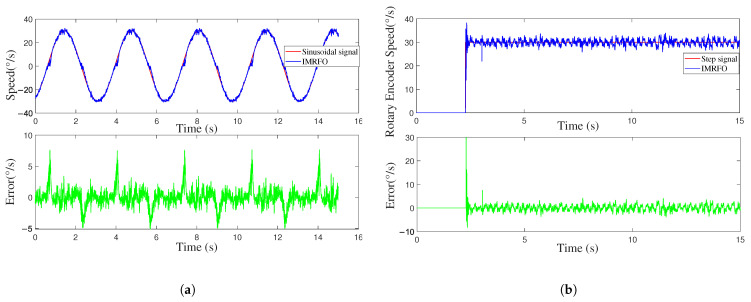
Experimental results under different input signals: (**a**) tracking curve and tracking error under sinusoidal signal and (**b**) tracking curve and tracking error under step signal.

Based on the training results of the IMRFO algorithm under sinusoidal signals, the optimal solution was determined as $K_{p}=1.053$, $K_{i}=12.6807$. Figure 12 shows the convergence curve of the fitness value, while Figure 13a,b presents the system outputs under sinusoidal and step signal inputs using the optimized control parameters.

**Figure 14:**
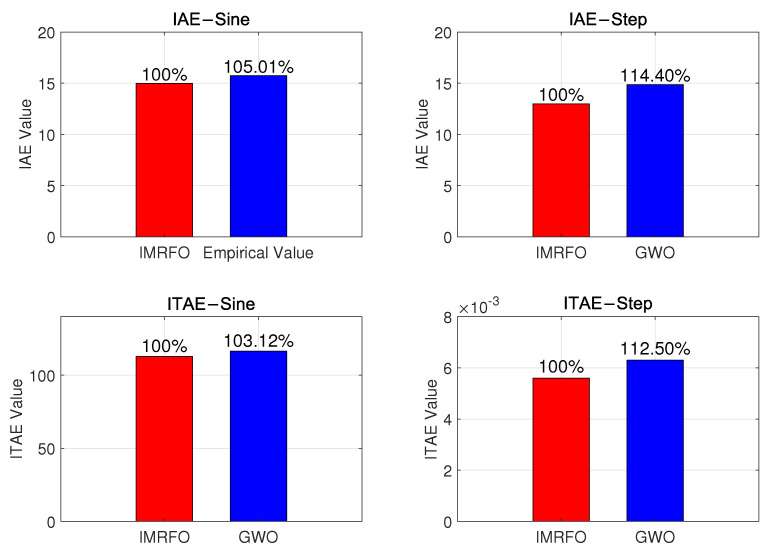
Comparison of performance metrics between parameter values optimized with IMRFO and empirical parameter values.

As shown in Figure 14, the optimized PI parameters generally outperform the empirically determined values under both sinusoidal and step signals. Under sinusoidal inputs, the optimized control system achieves lower IAE and ITAE values, resulting in a smoother response and smaller error. Under step inputs, the optimized control parameters again surpass the empirical values. Although the maximum overshoot increases slightly, both IAE and ITAE decrease significantly, demonstrating a notable improvement in error control.

## 5. Conclusions

This paper proposes a control parameter optimization method for PID control of artillery stabilization systems based on an improved version of the Manta Ray Foraging Optimization (MRFO) algorithm, which we call IMRFO. By introducing circle chaotic mapping to enhance population diversity and incorporating Lévy flight and a sigmoid function-based dynamic adjustment strategy to improve global search capability, the proposed IMRFO algorithm shows stronger performance on high-dimensional and multimodal optimization problems. Although the proposed IMRFO algorithm exhibits slightly slower convergence than standard MRFO on simple unimodal functions due to its increased exploration behavior, this tradeoff is beneficial in addressing complex control problems. Simulation results confirm that the optimized PID parameters significantly enhance the system’s steady-state accuracy and dynamic response. Compared with the conventional empirical tuning method, the proposed approach demonstrates superior robustness and control performance. 

## Figures and Tables

**Table 1:** Test functions.

Name	Function	Dimension	Range
Schwefel 2.22	$f_{1}=\sum_{i=1}^{n}\left|x_{i}\right|+\prod_{i=1}^{n}\left|x_{i}\right|$	30	[−10, 10]
Rosenbrock	$f_{2}=\sum_{i=1}^{n-1}\left(100{\left(x_{i+1}-x_{i}\right)}^{2}\right)+{\left(x_{i}-1\right)}^{2}$	30	[−30, 30]
Schwefel	$f_{3}=-\sum_{i=1}^{n}\left(x_{i}\sin\left(\sqrt{\left|x_{i}\right|}\right)\right)$	30	[−500, 500]
Penalized	$f_{4}=\frac{\pi }{n}10\sin^{2}\left(\pi y_{i}\right)+\sum_{i=1}^{n-1}\left({\left(y_{i}-1\right)}^{2}\left[1+10\sin^{2}\left(\pi y_{i}+1\right)\right]\right)+{\left(y_{n}-1\right)}^{2}+\sum_{i=1}^{30}u\left(x_{i},10,100,4\right)$	30	[−50, 50]
Penalized2	$f_{5}=0.1\left\{\sin^{2}\left(3\pi x_{1}\right)+\sum_{i=1}^{29}\left[{\left(x_{i}-1\right)}^{2}\left(1+\sin^{2}\left(3\pi x_{i+1}\right)\right)\right]\right\}+\left.{\left(x_{n}-1\right)}^{2}\left[1+\sin^{2}\left(2\pi x_{30}\right)\right]\right\}+\sum_{i=1}^{30}u\left(x_{i},5,100,4\right)$	30	[−50, 50]

**Table 2:** Parameter settings.

Algorithm	Parameter	Value
PSO	Inertia Weight	From 0.9 to 0.4 linearly
	Cognitive Coefficient	1.5
	Social Coefficient	1.5
GWO	Convergence Coefficient *a*	From 2 to 0 linearly
	Encircling Coefficient	2ar−a
	Spiral Constant	(a−1)·rand()+1
WOA	Linearly decreasing control factor *a*	From 2 to 0 linearly
	Encircling coefficient vectors	[−a,a]
	Prey influence coefficient vectors	[0,2]
ALO	Random walk boundary	lb/ub
	Antlion selection	roulette
CMA-ES	Initial step size σ	0.33×(Up−Low)
	Parent number μ	Pop/2
	Step control parameter $c_{s}$	(μ+2)/(Dim+μ+5)
	Covariance matrix update rate $c_{1}$	$2/({\left(\mathrm{Dim}+1.3\right)}^{2}+\mu )$

**Table 3:** Results of the six optimization algorithms on the test functions.

Function	Value	MRFO	IMRFO	PSO	GWO	WOA	ALO	CMA-ES
F1	Mean	**0**	**0**	2.07E −30	2.30E−107	5.91 E−234	1.72E−05	8.32E−13
	Best	**0**	**0**	1.73E−30	4.41E−109	1.65E−234	7.61E−06	6.68E−13
F2	Mean	**7.0550**	7.4650	21.8097	26.3748	21.7172	62.9911	29.9983
	Best	**6.0843**	6.4613	7.5990	25.9960	20.7771	17.0989	22.3406
F3	Mean	−8.99E+03	−1.02E+04	−6.71E+03	−3.01E+03	−8.36E+03	−4.76E+03	−6.75E+03
	Best	−1.00E+04	−1.13E+04	−7.98E+03	−3.72E+03	−9.00E+03	−5.09E+03	−8.28E+03
F4	Mean	0.0104	1.57E−32	0.0311	0.0530	3.80E−10	4.34E−11	4.24E−05
	Best	1.57E−32	1.57E−32	1.57E−32	0.0262	7.72E−11	9.66E−12	1.18E−16
F5	Mean	1.9174	0.0044	0.0033	0.2266	0.0183	2.4383	2.73E−16
	Best	1.35E−32	1.35E−32	1.35E−32	1.49E−06	6.29E−09	2.1674	1.57E−16

**Table 4:** Wilcoxon signed-rank test results: IMRFO vs. MRFO, PSO, and GWO.

Function	IMRFO vs. MRFO			IMRFO vs. PSO			IMRFO vs. GWO		
	**p-Value**	**W**	**Result**	**p-Value**	**W**	**Result**	**p-Value**	**W**	**Result**
f1	1.0000	0	=	0.0020	0	+	0.0020	0	+
f2	0.1309	43	=	0.0020	0	+	0.0020	0	+
f3	0.0020	0	+	0.0020	0	+	0.0020	0	+
f4	1.0000	0	=	0.1250	0	=	0.0020	0	+
f5	0.0039	0	+	1.0000	9	=	0.0020	0	+

**Table 5:** Wilcoxon signed-rank test results: IMRFO vs. WOA, ALO, and CMA-ES.

Function	IMRFO vs. WOA			IMRFO vs. ALO			IMRFO vs. CMA-ES		
	**p-Value**	**W**	**Result**	**p-Value**	**W**	**Result**	**p-Value**	**W**	**Result**
f1	0.0020	0	+	0.0020	0	+	0.0020	0	+
f2	0.0020	0	+	0.0020	0	+	0.0020	0	+
f3	0.0020	0	+	0.0020	0	+	0.0020	0	+
f4	0.0020	0	+	0.0020	0	+	0.0020	0	+
f5	0.0195	5	+	0.0020	0	+	0.5371	34	=

**Table 6:** System structural parameters.

Structure	Name	Value
Turret Launching System	Turret Mass (kg)	5517
	Launching System Mass (kg)	80
	Turret Rotation Radius (m)	1.1
	Barrel Length (m)	5.3
Electric Cylinder Installation Parameters	Initial Angle of Electric Cylinder (°)	arcsin(0.6)
	Initial Length of Electric Cylinder (m)	0.6
	Distance from Ear to Electric Cylinder Drive Point (m)	0.8

## Data Availability

The data and code of the current study can be obtained from the corresponding author upon reasonable request.

## DURC Statement

The current research is limited to the field of intelligent control and optimization algorithms, which are primarily used to improve the performance of control systems. This work does not pose a threat to public health or national security. The authors acknowledge the dual-use potential of optimization technologies and confirm that all necessary precautions have been taken to prevent potential misuse.
